# Disjunct distribution of *Hesperotettixspeciosus* (Orthoptera, Acrididae) from the Black Belt Prairie of Alabama, United States

**DOI:** 10.3897/BDJ.12.e133499

**Published:** 2024-09-12

**Authors:** Shelby Grice, JoVonn G. Hill

**Affiliations:** 1 Mississippi State University, Mississippi State, United States of America Mississippi State University Mississippi State United States of America

**Keywords:** Western green grasshopper, showy grasshopper, blackland prairie, state record

## Abstract

Inventories of the flora and insect fauna of the Black Belt Prairie of Mississippi and Alabama have documented disjunct populations of species broadly distributed in the Great Plains, including two grasshopper species. During a recent survey of a large privately-owned prairie remnant in Sumter Co., Alabama, we documented a disjunct population of a third grasshopper species — *Hesperotettixspeciosus*, extending the known distribution of this species significantly eastwards. The discovery of this population is surprising due to the number of previous survey efforts of the Black Belt, Alabama and Mississippi overall and exemplifies the importance of conservation on private lands.

## Introduction

The Black Belt Prairie is a crescent-shaped region that extends from McNary County in southern Tennessee through east-central Mississippi to Russell County, Alabama, near the Georgia border (Lowe 1921, Smith 1926, Stephenson and Monroe 1940, Barone 2005). The Black Belt is characterised by calcareous loamy soils and the presence of distinct grassland flora (Barone and Hill 2007, Campbell and Seymour 2014). The flora, combined with studies of insects, suggest a prehistoric connection to the Great Plains and its role as a refugium for prairie life during the last glacial period (Brown 2003, Hill and Brown 2010, Hill and Barone 2018). The Black Belt is underlain by Cretaceous age Selma chalk that is composed of fossiliferous, soft, white-grey limestone. Selma chalk weathers into the fertile black soil for which the region is named (Logan 1903, Lowe 1921, Stephenson and Monroe 1940, Hicks and Haynes 2000). Floristic surveys of Black Belt prairie remnants have documented a distinct plant community similar to the eastern tall-grass prairies of the Great Plains, with several rare or critically imperilled plants present (Schuster and McDaniel 1973, Leidolf and McDaniel 1998, Barone and Hill 2007).

General Land Office surveys stated that prairies covered at least 144,000 hectares of the Black Belt in the 1830s. Since then, more than 99% of those prairies have been lost to both agricultural and urban development (Noss et al. 1995). Most notably, development of cotton farms in the mid-1800s to early 1900s destroyed much of the native vegetation, leaving behind patchy areas of old fields, pastures and occasional clusters of forest alongside streams (Barone 2005). Remnants still intact are threatened by anthropogenic (human) development, erosion and the spread of *Juniperusvirginiana* L. (Cupressaceae) (Eastern Red Cedar) due to fire suppression in these areas (Evans et al. 1989, Hill and Barone 2018). The Mississippi Natural Heritage programme gives the Black Belt Prairie a ranking of S1, meaning that the remnants are “critically imperilled” in the State because of their rarity, as well as their vulnerability to extirpation (The Nature Conservancy 2023).

The insect fauna of the Black Belt Prairie has been surveyed by staff of the Mississippi Entomological Museum (MEM) since the late 1980s. These surveys have documented populations of insects disjunct from the Great Plains, such as the bee *Tetraloniellaalbata* (Cresson) (Anthophoridae), grasshoppers such as *Pseudopomalabrachyptera* (Scudder) (Acrididae) and *Campylacanthaolivacea* (Scudder) (Acrididae), Cerambycidae such as *Tetraopestexanus* Horn, as well as several species of moths, all of which emphasise the significance of the region's biodiversity (MacGown and Schiefer 1992, Schiefer 1998, Brown 2003, Hill 2005, Hill 2007, Hill and Brown 2010).

Recently, MEM staff have been conducting insect surveys at a high-quality Black Belt remnant in Sumter County, Alabama. From here, we have discovered a population of *Hesperotettixspeciosus*, commonly known as the showy grasshopper (Fig. 1). This represents the third grasshopper species with a population normally found in the Great Plains, now found in the Black Belt and a new State record for Alabama (Dakin and Hays 1970, Hill 2005).

**Figure 2.**
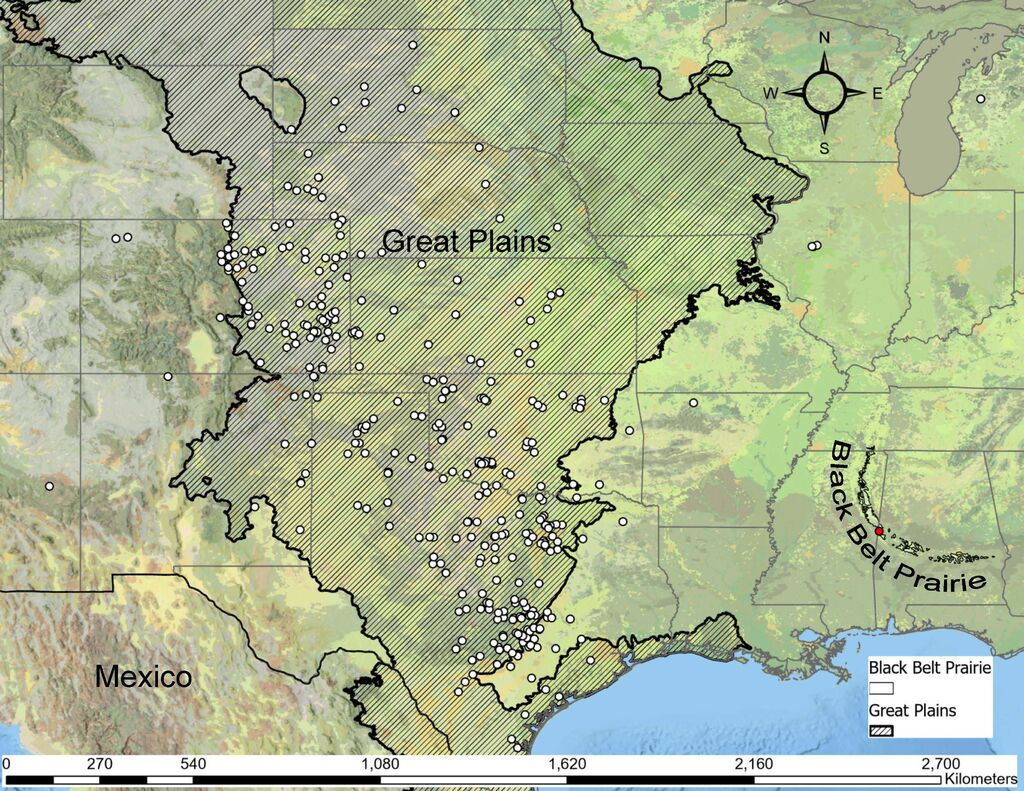
A map of the United States and Mexico, highlighting the Great Plains and the Black Belt Prairie, with white points showing occurrence records of *Hesperotettixspeciosus*, as well as a red point showing the new occurrence from Sumter Co., Alabama.

*Hesperotettixspeciosus* is expected in the midwestern great plains of the United States, where prairies are abundant (Fig. 2). This species feeds on various species of sunflower (*Helianthusannuus* & *H.petiolaris*), marshelder (*Ivaxanthifolia*) as well as ragweed (*Ambrosiapsilostachya*) (Mulkern 1969). This species of grasshopper is identifiable by its bright purplish-red stripe that occurs along the median of the pronotum as well as a similarly-coloured stripe on the dorsal edge of the hind femora (Blatchley 1920). This discovery represents a significant disjunct population much further southest than expected (Dakin and Hays 1970, Hill and Barone 2018) and may offer opportunities to better understand the biogeographic relationship between the Great Plains and the Black Belt Prairie.

## Methods

**Figure 3.**
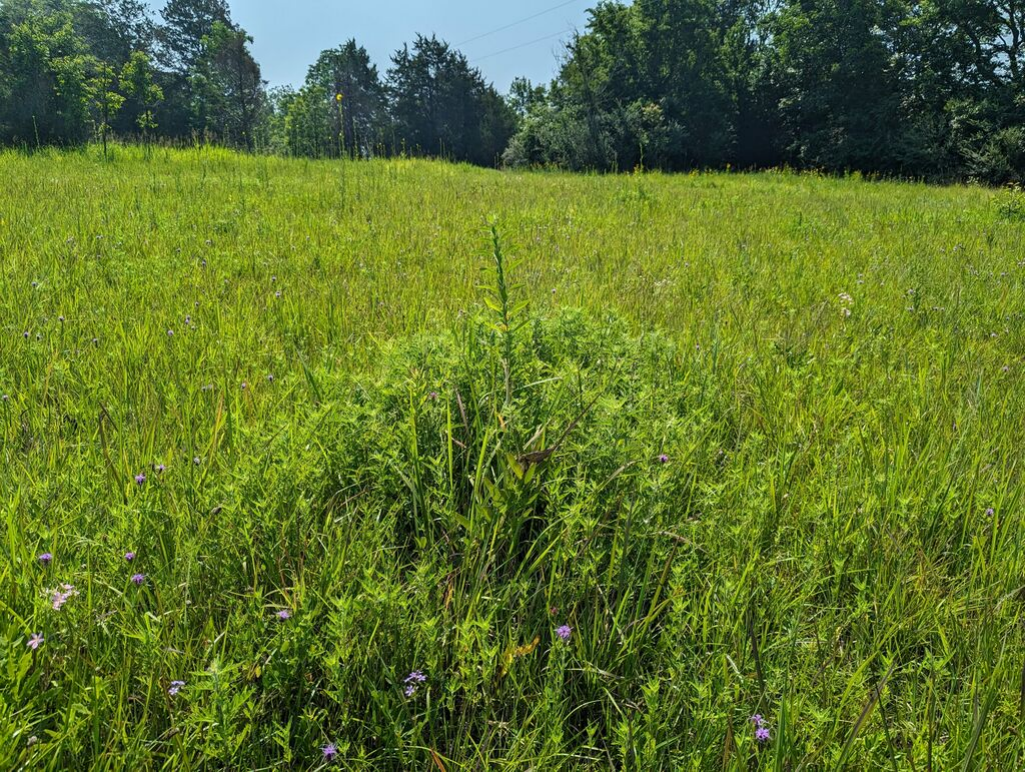
Field site with western ragweed (*Ambrosiapsilostachya*), Wildhorse Prairie.

Entomologists from the Mississippi Entomological Museum arrived at the private property site at mid-day and began walking through the tall grassland, looking for grasshoppers of interest. It is known that *Hesperotettixspeciosus* eats plants within the Asteraceae family, so the entomologists referred to previous floral surveys previously done in the area (Leidolf and McDaniel 1998, Barone and Hill 2007, Hill and Barone 2019), where it has been documented that the prairie is largely dominated by grasses (Poaceae) with asters dotting the landscape (Fig. 3). Specimens were collected on *Ambrosiapsilostachya* DC. using a standard size sweep net (30-cm diameter). The entomologists on foot used a “flush and capture” method whereby grasshoppers were collected with a net as they dispersed away from the approaching collectors. This “flush and capture” method is the most common means of conducting grasshopper species-composition studies (Capinera and Sechrist 1982, Evans 1989, Kemp et al. 1990, Squitier and Capinera 2002). Specimens were euthanised with either 100% ethanol or potassium cyanide on site. The specimens euthanised in potassium cyanide, once taken to the lab, had internal genitalia extruded and compared to specimens of the same species from their known distribution. These identified specimens were then pinned and labelled. One hind leg from each individual stored in ethanol has been sent for sequencing as part of a larger Melanopline population genetic study. Voucher specimens are deposited in the Mississippi Entomological Museum.

## Results

Ten individuals were collected on 29 June 2023 near Geiger, Alabama by the authors and Jireh Mwamukonda. The specimens reported were found associated with western ragweed (*Ambrosiapsilostachya*), whose Black Belt populations are also considered disjunct. Two males and two females were euthanised in potassium cyanide and then pinned for preservation (Fig. 4), in addition to two males and four females collected in 100% ethanol for DNA preservation.

During pinning, the internal male genitalia of one specimen was extruded, revealing shape of the epiphallus and the aedeagus for later morphological comparisons to males from mid-western populations (Figs 5, 6).

The reported distribution of this species is from Montana east to Minnesota and south to New Mexico and Texas (Vickery and Kevan 1985). In Nebraska, it is consistently present in the sandhills, xeric soils and somewhat less common, but present in rangeland on loess soils in the central plains region of the United States (Vickery and Kevan 1985). The wings of *Hesperotettixspeciosus* are shorter than the abdomen; therefore, it is presumably unable to fly for great lengths (especially the more robust females). As such, the dispersal of this species is likely very limited. This newly-discovered Alabama population of *H.speciosus* is significant because the furthest east that this species was found previously was nearly 645 km away in Boone County, Arkansas. There have been outliers in areas such as Lake County, Michigan and Mason County, Illinois, but they previously had not been recorded as far south as Alabama until now.

## Discussion

The Mississippi Entomological Museum has conducted numerous surveys within Black Belt Prairie remnants over the last thirty years. Despite historical state-wide grasshopper surveys by Dakin and Hays (1970), as well as surveys targeting Black Belt Prairies by Hill (2007), *H.speciosus* had remained undiscovered until now. This new population could indicate movement of small groups from their normal range in the Great Plains into areas further south and east from populations with longer than average wing lengths. However, it is more likely that this is a relic population from the Pleistocene when the southeast was drier and grasslands were more expansive, allowing western biota to expand into the region (Brown 2003, Hill and Barone 2018). After further climactic shifts, the populations would have lost connecting micro-habitats and become isolated and, without strong and consistent dispersal capabilities, the isolation has persisted.

Our discovery presents an advancement in our understanding of the biodiversity and the ecological complexity of the Black Belt prairie and contributes valuable data to our understanding of the biogeography of grasslands in the south-eastern North America. Just as Aldo Leopold (1972) stated, "To keep every cog and wheel is the first precaution of intelligent tinkering". Such findings underscore the critical importance of conserving these isolated remnants of south-eastern grasslands as even small pockets of a specific habitat can harbour species not known or presumed extirpated from a region that can help us understand the ecology and biological history of the community. This discovery also speaks of the uniqueness of Wildhorse Prairie, the importance of conservation on private lands and the care and management practices used by the landowners for over 100 years. Preserving each south-eastern prairie remnant is crucial to safeguard the array of disjunct species thriving within these small habitats.

## Figures and Tables

**Figure 1a.**
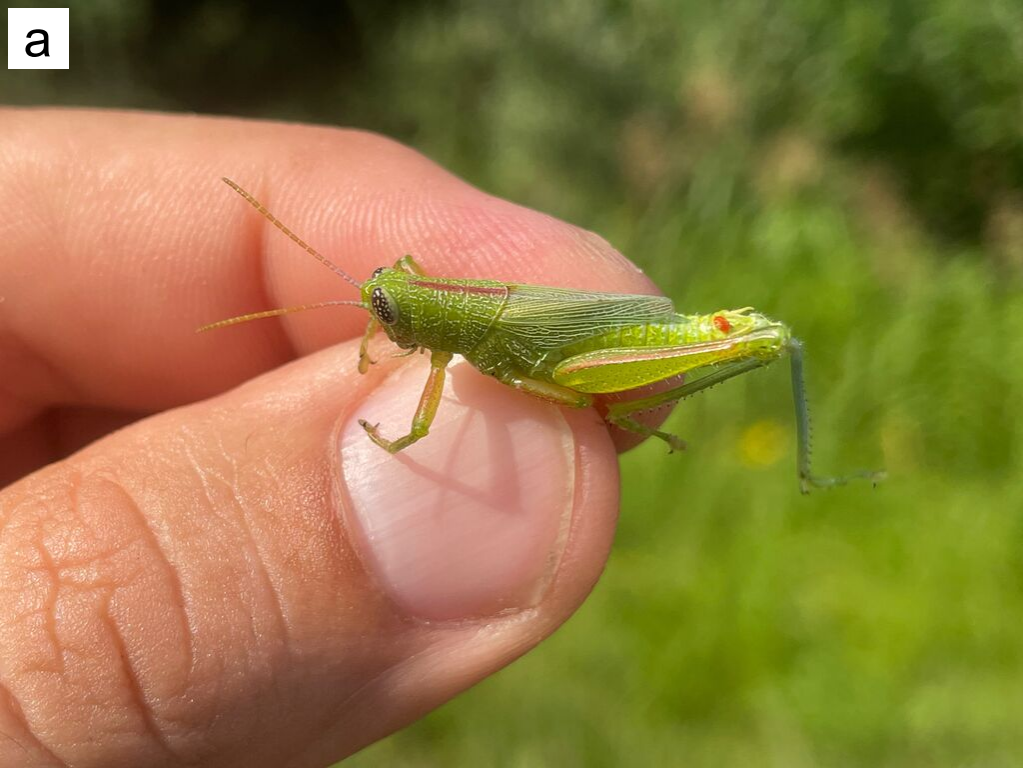
*Hesperotettixspeciosus* male;

**Figure 1b.**
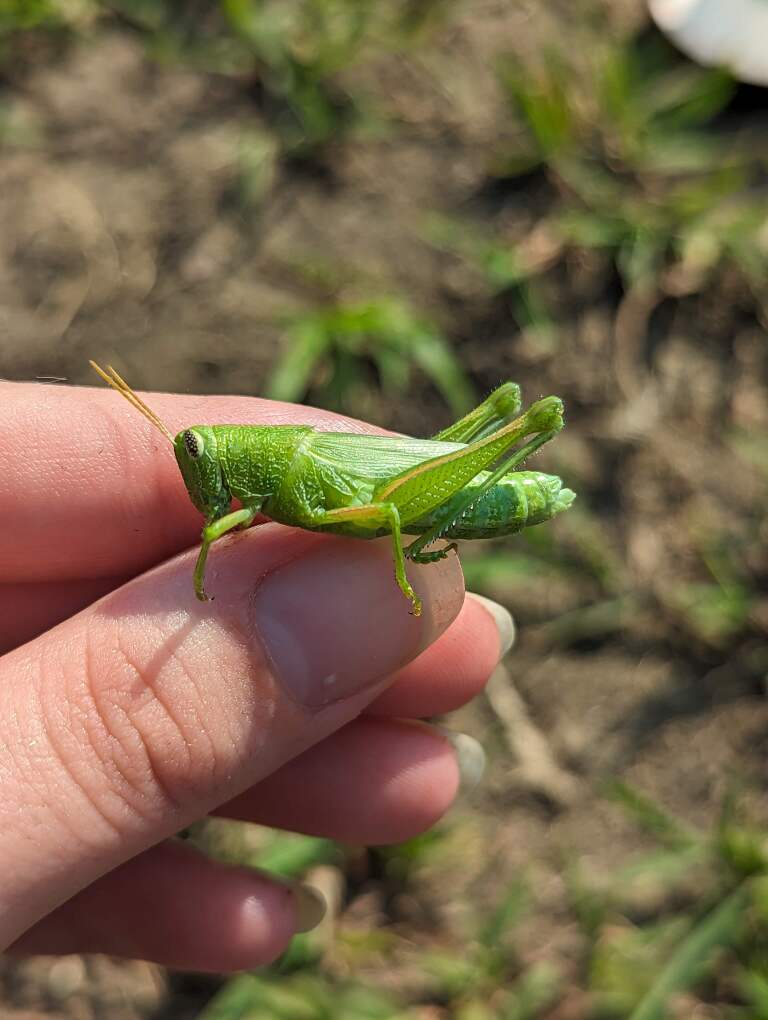
*Hesperotettixspeciosus* female.

**Figure 4a.**
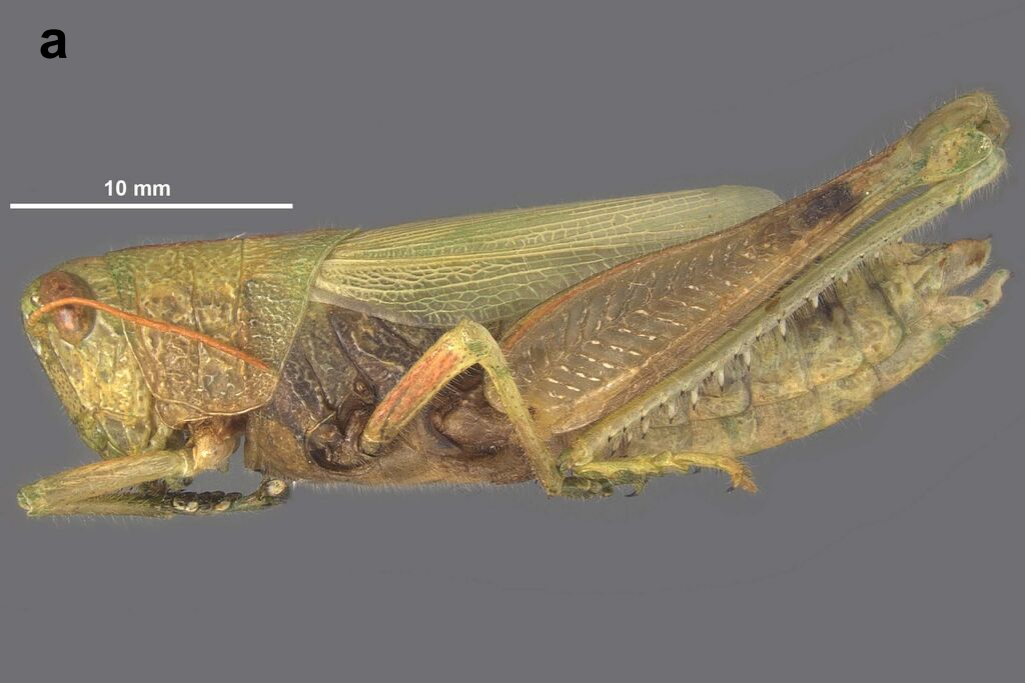
*Hesperotettixspeciosus* Female Habitus, scale bar 10 mm;

**Figure 4b.**
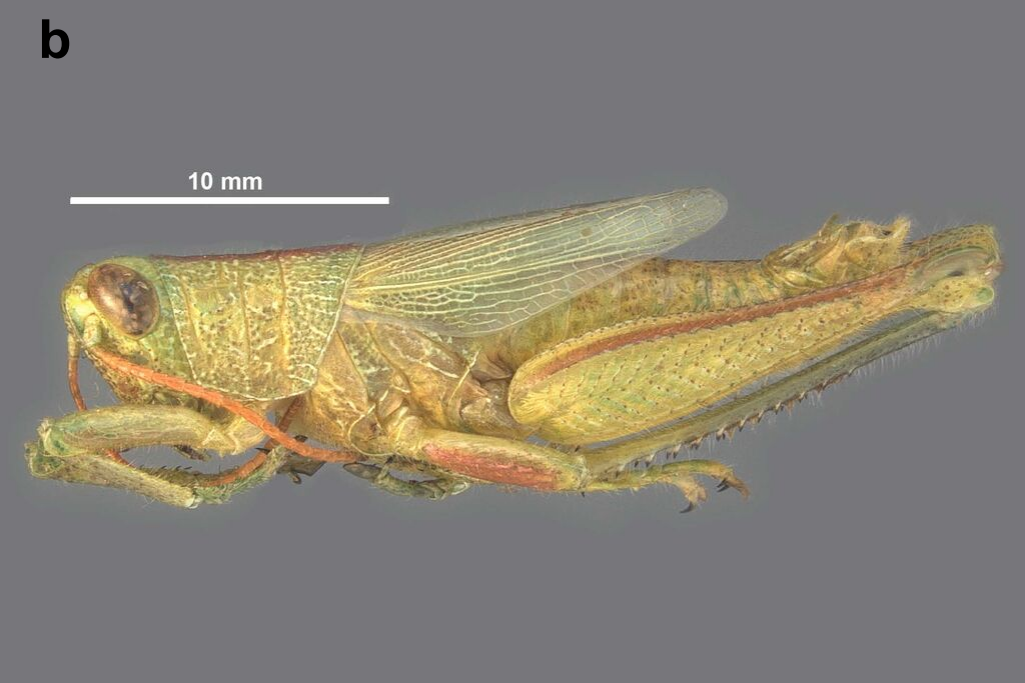
*Hesperotettixspeciosus* Male Habitus, scale bar 10 mm.

**Figure 5a.**
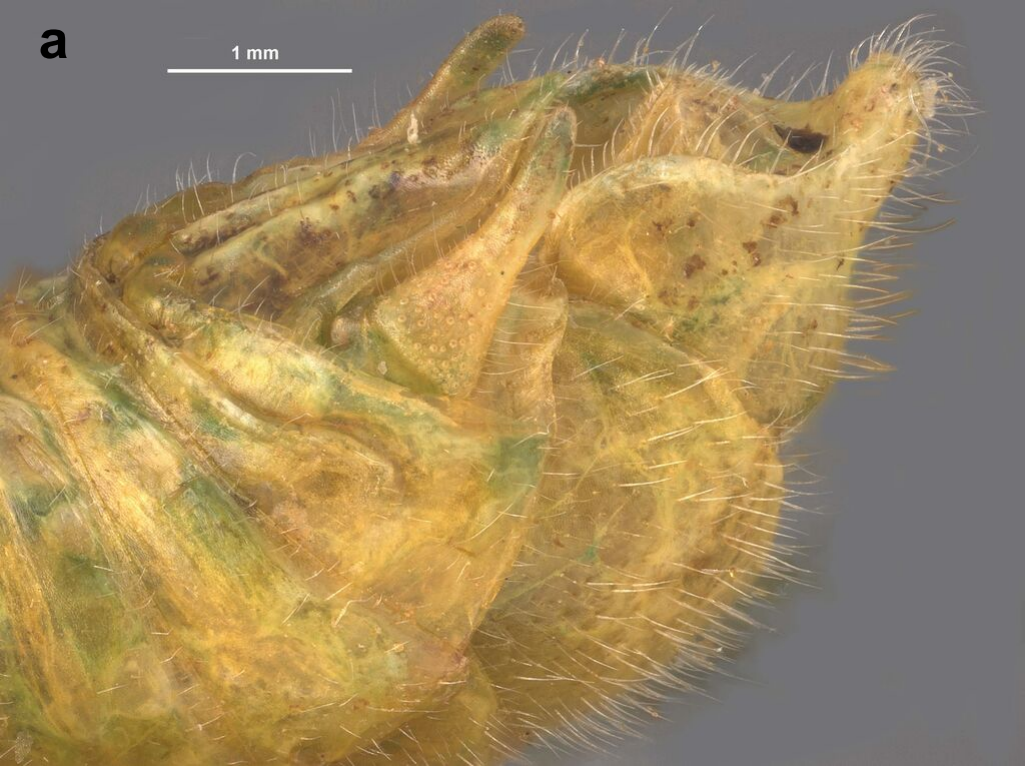
Lateral image taken of the terminalia of a male *Hesperotettixspeciosus*, scale bar 1 mm;

**Figure 5b.**
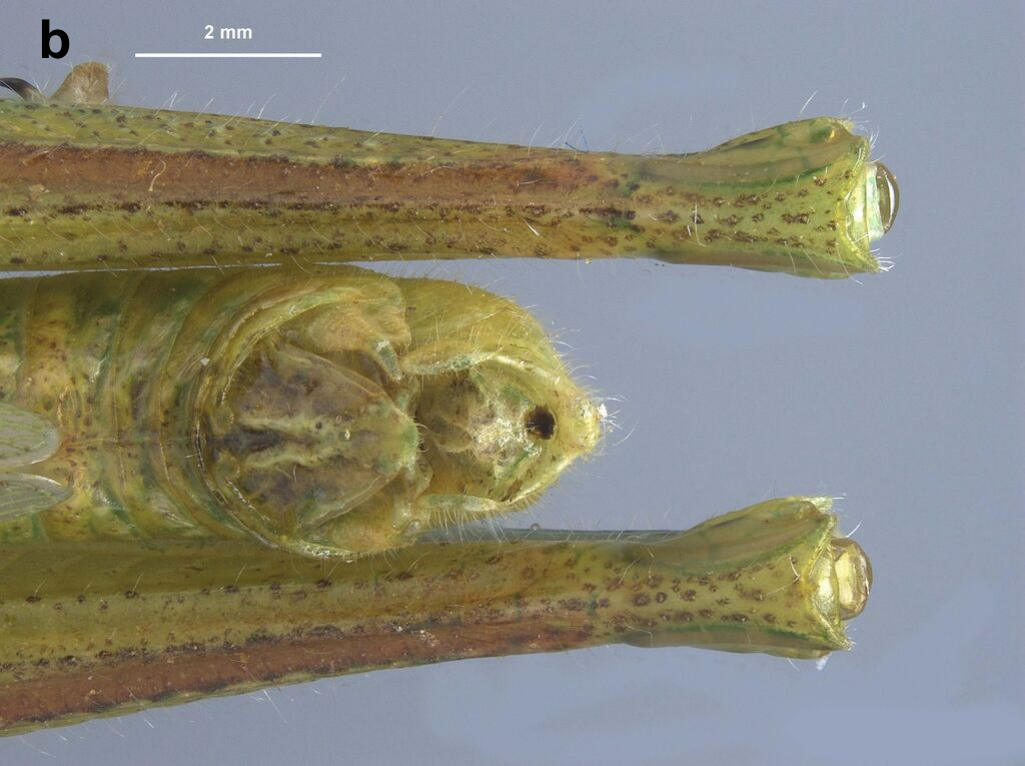
Dorsal image of the male terminalia, scale bar 2 mm;

**Figure 5c.**
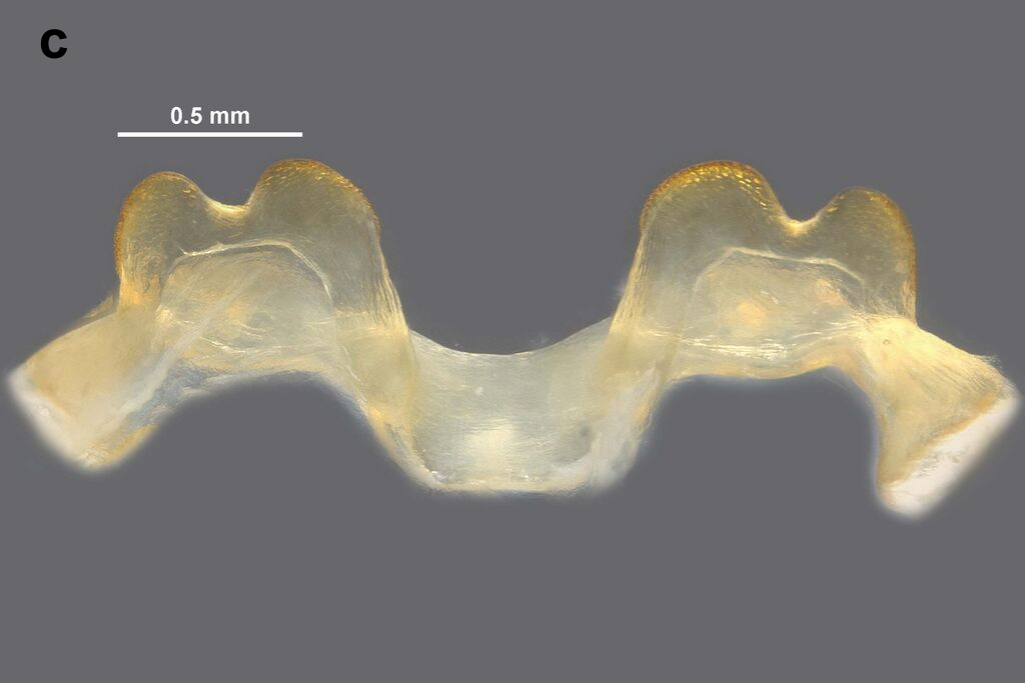
Caudal view of the epiphallus, scale bar 0.5 mm;

**Figure 5d.**
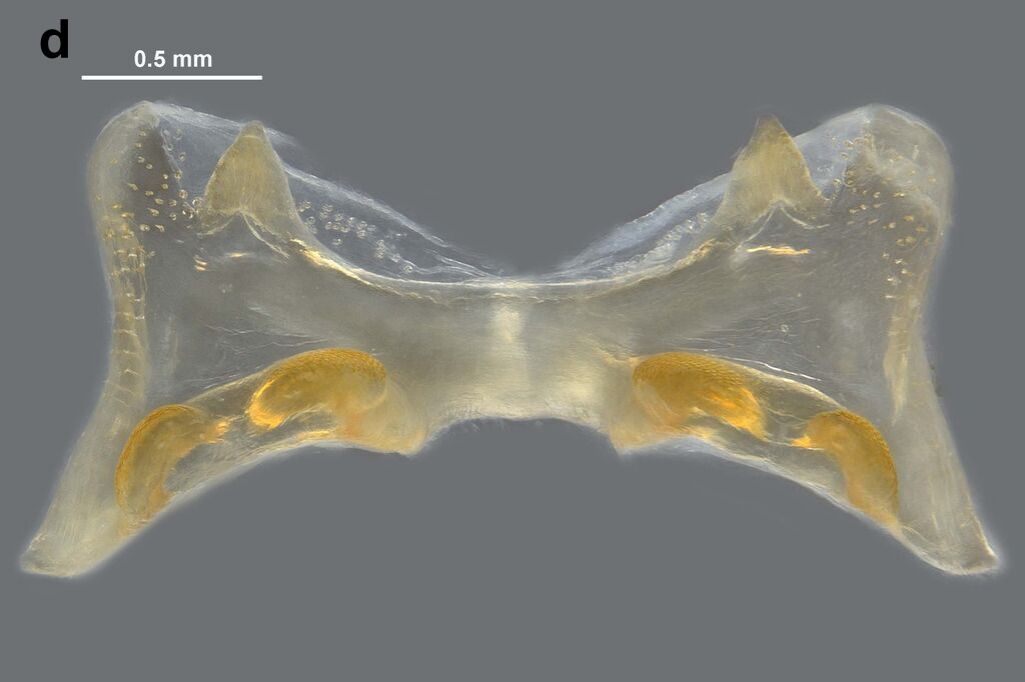
Dorsal view of the epiphallus, scale bar 0.5 mm.

**Figure 6a.**
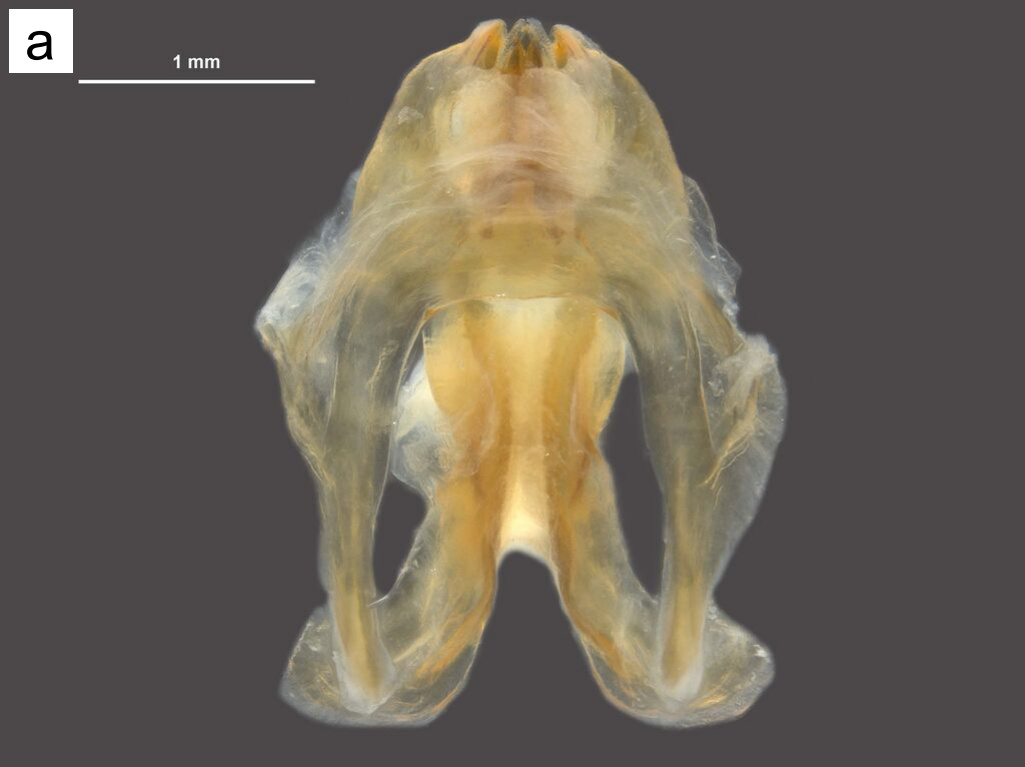
Dorsal view of *Hesperotettixspeciosus* phallic complex, scale bar 1 mm;

**Figure 6b.**
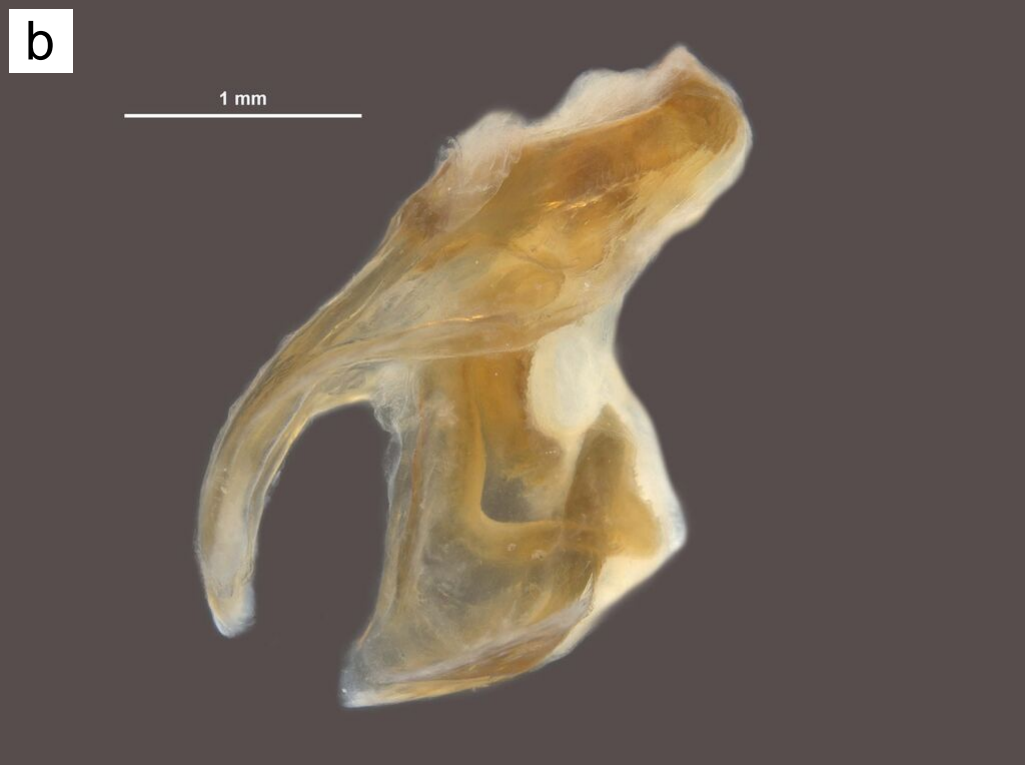
Lateral view of the phallic complex, scale bar 1 mm;

**Figure 6c.**
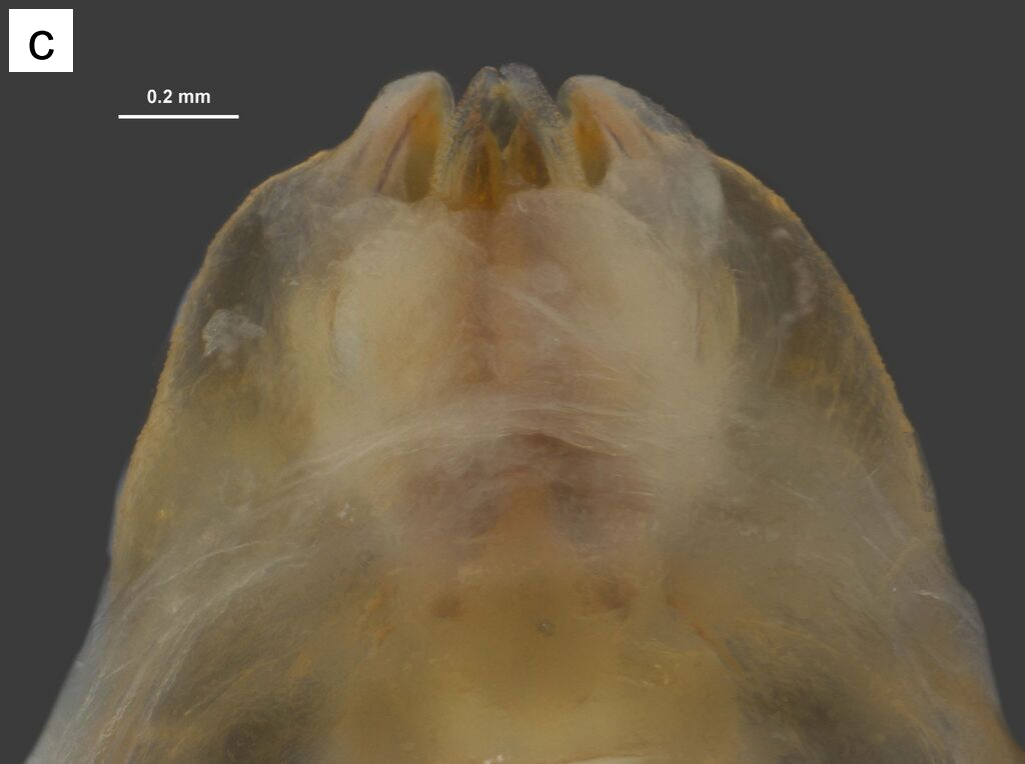
Dorsal view of aedeagus, scale bar 0.2 mm;

**Figure 6d.**
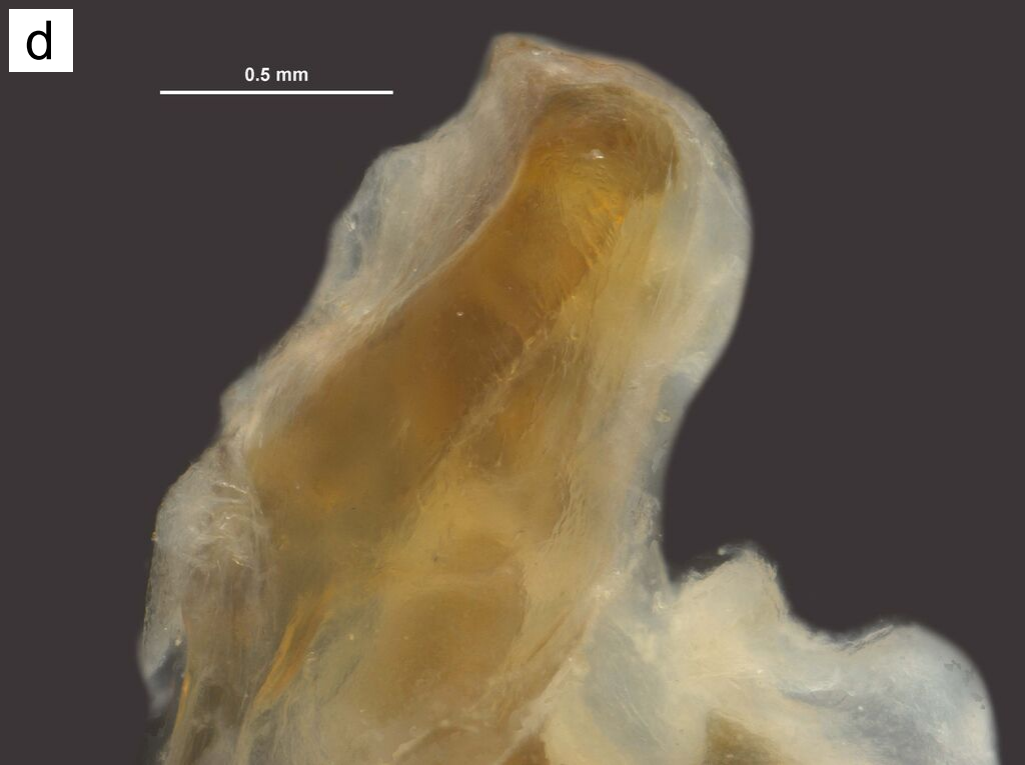
Lateral view of aedeagus, scale bar 0.5 mm;

**Figure 6e.**
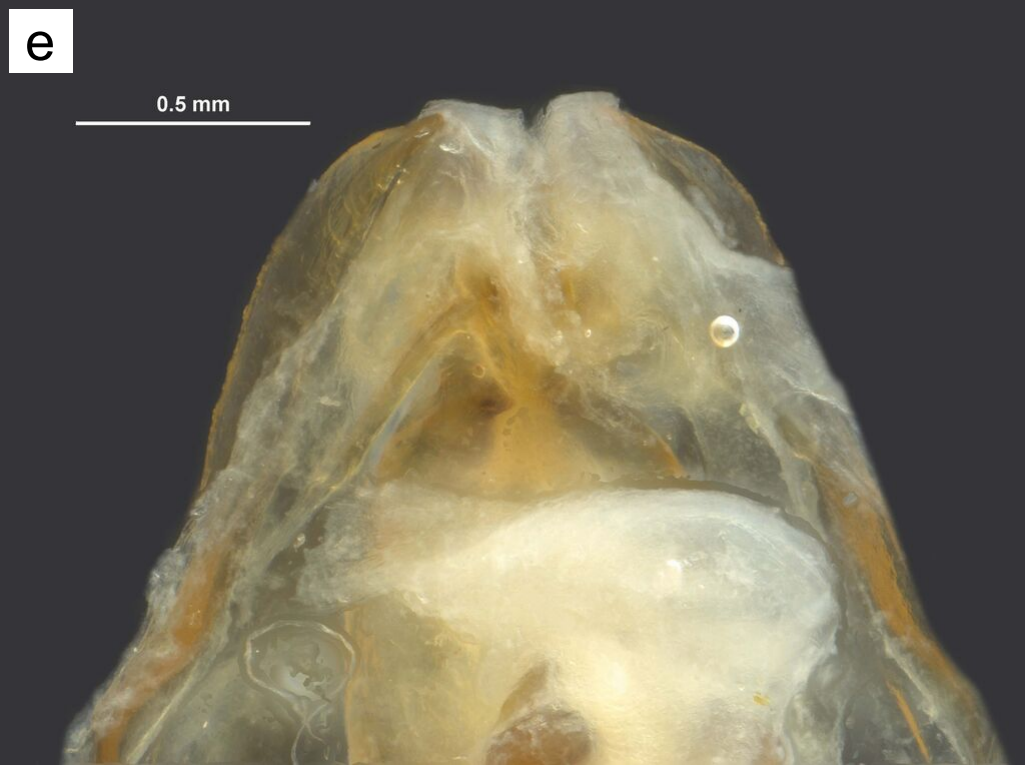
Caudal view of aedeagus, scale bar 0.5 mm.
